# Mechanical Behavior of Printed Strain Hardening Cementitious Composites

**DOI:** 10.3390/ma13102253

**Published:** 2020-05-14

**Authors:** Stefan Chaves Figueiredo, Claudia Romero Rodríguez, Zeeshan Y. Ahmed, Derk H. Bos, Yading Xu, Theo M. Salet, Oğuzhan Çopuroğlu, Erik Schlangen, Freek P. Bos

**Affiliations:** 1 Microlab, Faculty of Civil Engineering and Geosciences, Delft University of Technology, 2628 CN Delft, The Netherlands; C.RomeroRodriguez@tudelft.nl (C.R.R.); Y.Xu-5@tudelft.nl (Y.X.); o.copuroglu@tudelft.nl (O.Ç.); Erik.Schlangen@tudelft.nl (E.S.); 2 Department of the Built Environment, Eindhoven University of Technology, 5600 MB Eindhoven, The Netherlands; Z.Y.Ahmed@tue.nl (Z.Y.A.); d.h.bos@tue.nl (D.H.B.); t.a.m.salet@tue.nl (T.M.S.); f.p.bos@tue.nl (F.P.B.)

**Keywords:** strain hardening cementitious composites (SHCC), engineered cementitious composites (ECC), 3D printing, fibre reinforcement

## Abstract

Extrusion based additive manufacturing of cementitious materials has demonstrated strong potential to become widely used in the construction industry. However, the use of this technique in practice is conditioned by a feasible solution to implement reinforcement in such automated process. One of the most successful ductile materials in civil engineering, strain hardening cementitious composites (SHCC) have a high potential to be employed for three-dimensional printing. The match between the tailored brittle matrix and ductility of the fibres enables these composites to develop multiple cracks when loaded under tension. Using previously developed mixtures, this study investigates the physical and mechanical performance of printed SHCC. The anisotropic behavior of the materials is explored by means of mechanical tests in several directions and micro computed tomography tests. The results demonstrated a composite showing strain hardening behavior in two directions explained by the fibre orientation found in the printed elements. Moreover, the printing technique used also has guaranteed an enhanced bond in between the printed layers.

## 1. Introduction

The additive manufacturing of cementitious materials (AMoC) is rising as one of the solutions to achieve fully automated building processes. One of the most rapidly spreading technologies is extrusion-based deposition of subsequent layers, popularly known as 3D concrete printing (3DCP) [1]. In 3DCP, printable mortars usually have a dough-like consistency in a single stage mixing process, or in a double stage process in which accelerators or viscosity modifiers are added at or near the printing head. In the fresh state, the printed material should be able to resist their self-weight, as well as the loads caused by additional layers. Many publications have already testified the success and potentials of this new construction technique [2,3,4,5]. Different types of materials have been used to print such as geopolymers [6], calcium aluminate cement [7], calcined clay cement [8,9], Portland cement with copper tailing [10], and many other examples. The combination of an automated deposition with tailored mix-designs guarantees the freedom of form usually reported in studies in the field of 3DCP [11,12,13].

As cementitious materials exhibit brittle failure behavior, their application in construction requires a kind of reinforcement to ensure structural safety. In conventional cast concrete, reinforcement with steel bars is generally used, as well as some other strategies such as pre-stressed reinforcement, fibre reinforcement, or a combination thereof. These solutions are not necessarily applicable to 3DCP, as they may interfere with the manufacturing process, limit the geometrical freedom presented by 3DCP, produce insufficient residual strength, or apply only to rather specific situations. As it is generally recognized, the lack of suitable reinforcement methods could seriously hinder the potential applicability of the technology [14,15], several options are being developed by different groups. Until now, only two strategies have been presented that are fully integrated with the 3DCP process: the automated entrainment of reinforcement cable [16,17,18,19], and the application of fibres which will be further discussed in this manuscript.

Several studies have already investigated strategies to reinforce extruded cementitious composites, a process similar to 3DCP, as demonstrated by Shen et al. [20] with layered elements with different PVA fibre content or using natural fibres [21]. Fibre reinforcement for printed concrete was initially studied by Hambach and Volkmer [22]. They tested not only different fibres (carbon, glass, and basalt) to reinforce a blended cement paste with silica fume but also different printing patterns. Other studies assessed the influence of various fibre types on mechanical properties, such as the tensile and compressive strengths, the interlayer strength, and ductility [23,24,25,26,27]. A dominant orientation of the fibres in the flow direction is commonly reported. In this direction, tensile strength is found to be higher [22,23,27]. An important aspect to consider is whether the applied fibres are compatible with the 3DCP equipment that is used.

A special family of fibre reinforced mortars are the Strain-Hardening Cementitious Composites (SHCCs, in literature also referred to as Engineered Cementitious Composites, ECCs). This type of material is able to absorb significantly higher amounts of energy during failure by tensile forces in comparison to a conventional fibre reinforced material. The strain hardening performance is obtained by tailoring the bond strength between the fibre and the matrix. This behavior enables this type of composite to develop a very small and well distributed cracking pattern [28,29]. This results in superior behavior in terms of mechanical performance [30], durability [31], and when employed as a repair mortar [32].

Therefore, SHCC has significant potential in automated concrete manufacturing. Some research has already reported the use of this material in the development of mixtures to be applied in 3D printing of cementitious composites. In one of the first published investigations regarding the printability of SHCCs, fresh state properties were assessed when some of the constituents of the matrix were changed [7]. As well as reported in investigations of ordinary SHCCs [33,34], a printable version of the composite [7] is also usually reinforced by 2% by volume of PVA fibres (12 mm length and 40 μm diameter). With the exception of reference mixtures, 0.4 wt% of hydroxypropyl methylcellulose (HPMC) was used as a rheology modifier. To increase the hardening speed, some of the evaluated mix-designs contained calcium aluminate cement and silica fume as well. A manual caulk–gum apparatus was used to print specimens out of selected mixtures. Some of the authors’ conclusions stated that calcium aluminate cement has effectively accelerated the hardening, which positively contributed to the buildability of this type of material. The mixing parameters such as water temperature and batch volume influenced significantly the rheological properties and therefore must be controlled to achieve reproducible printing results. Finally, modifications of the SHCCs mix-design, to facilitate the printing process, did not affect the strain-hardening properties of the composite (around 4% maximum strain capacity). In a subsequent study, printed ECC with varying fibre content per layer was presented as a strategy to optimize fibre usage [35]. In another study, high ductility was found when the use of different content of Polyethylene fibre reinforcement with additions of sulphoaluminate cement to improve buildability was investigated [36].

The anisotropic stress–strain behavior of a PVA reinforced SHCC was the subject of a study performed by Yu and Leung [37]. Remarkably, they reported limited strain-hardening performance when load was applied perpendicularly to the interfaces. Nevertheless, high ductility was found in other loading directions.

Another investigation on printable SHCCs was presented by Ogura et al. [38], who used an automated printing setup based on a moving printing head connected to a progressive cavity pump. An extensive experimental investigation was performed with mix-designs composed of CEM II/A-M (S-LL) 52.5R, silica fume, fly ash, sand of maximum grain size of 1 mm, and high-density polyethylene microfibres (HDPE). Three different reinforcement levels were investigated (0.3, 1, and 1.5 vol.%) along with three sand-to-binder ratios and two water-to-binder ratios. Extrudability tests were performed using a ram-extruder and the flowability was measured by means of a standardized flow test. Composite’s mechanical performance was assessed by means of direct tensile and compressive tests to compare printed and cast specimens. The authors showed that the extrusion force strongly depends on the proportion of raw materials. The total volume of fibres was not a crucial factor in this property, but a sand-to-binder ratio has played an important role. Results pointed out that using a smaller amount of sand in the mixtures usually led towards lower extrusion forces due to increased cohesion. Moreover, the mechanical properties of printed materials were also improved. Probably due to the preferential alignment of the fibres in printed samples, the total strain capacity of the composites reinforced with 1.5 vol.% were significantly improved. All tested specimens delivered strain-hardening behavior with multiple small cracks.

An interesting use of the anisotropic behavior that extrusion based materials own were reported by [39,40]. These studies have demonstrated, not necessarily using cementitious materials that the different mechanical properties for different directions can be used to develop larger scale materials with enhanced properties and optimized performance.

The study reported in [41] has extensively evaluated the rheological parameters involved in the development of printable SHCCs. Several modifications in the mix-design of such composite were investigated using a ram-extruder coupled with the Benbow–Bridgewater model [42]. With the developed methodology, a relation of the rheological properties of printable materials with their ability to possess adequate printability and buildability was achieved. From that research, two outstanding mixtures were selected to be used in the current study.

The aim of this study was to give a comprehensive evaluation of physical and mechanical properties of SHCC especially tailored to be 3D printed in a larger scale than those previously discussed. As it was expected that the extrusion of the material might generate preferential orientation of fibres and consequently anisotropy, a protocol was followed to characterize the composite. Non-destructive techniques were employed to study the air void content and fibre orientation. Particularly, the interface region between layers was of interest. In addition, a range of destructive mechanical tests was performed to determine compressive, tensile, and flexural strength and toughness. Various loading directions (relative to the print path) were assessed, as well as 2 print layer interval times and beams with one to four layers height (for flexural bending).

## 2. Experimental Methods

### 2.1. Materials and Methods

Two types of SHCC were evaluated. They were developed based on [33,34]. Their rheological properties were explored in an earlier publication [41]. In that study, the influence of water, superplasticizer, and viscosity modifier admixture (HPMC) contents, as well as the fibre volume and sand grain size distribution on the rheology of theses mixtures were evaluated. As it was reported, only one mix-design of each SHCC was chosen to be printed. They had the best initial shear and bulk yield stresses along with a successful printability tests. These two rheological properties were considered essential to extrude fibre reinforced cementitious composites. In Table 1, the composition of each mixture was given. The water-to-solid ratio, as well as the total amount of chemical admixtures used in both mixtures was crucial to achieve the desired rheological properties. The initial bulk and shear yield stresses of both mixtures were (34.74 ± 5.20) kPa and (4.41 ± 0.18) kPa for XVA3PVA20 and (34.26 ± 1.98) kPa and (2.49 ± 0.08) kPa for YVA4PVA20-S05, respectively. Physical and chemical characteristics of the raw material used can be found in in [41].

A total volume of 50 L of SHCC was prepared. Due to high viscosity in the fresh state of both mixtures, two batches of 25 L were mixed in a planetary mixer. The equipment used for mixing is a TMV 75 pan mixer manufactured by Van der Zalm Nuth B.V., with a total capacity of 75 L, an engine power of 2.2 kW, and a single speed. A similar mixing procedure as the one reported in [41] was used. In summary, the following steps were executed:

- All dry materials were mixed for two minutes;
- While mixing and within one minute, all the water mixed with superplasticizer was added to the dry mix;
- The last step from the mixing was used to make sure that a homogeneous fresh material was achieved, with no fibre lumps or dry powder left behind.

**Figure 1:**
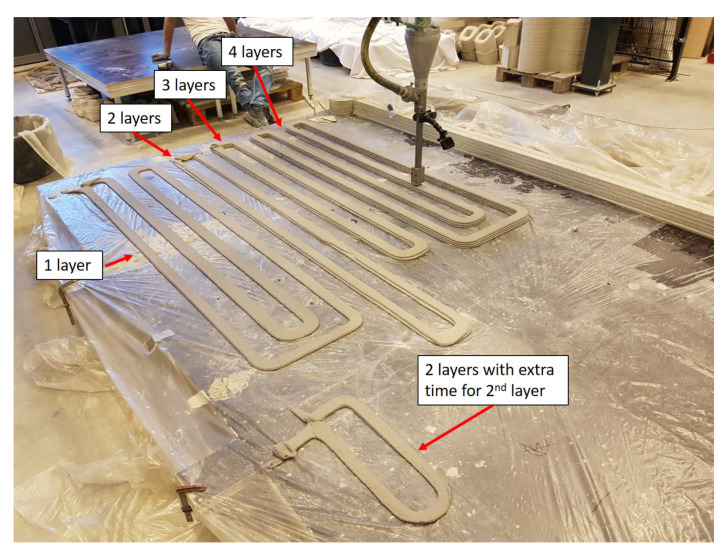
Printing table with final printed elements with one, two, three, and four layers.

The material was printed at the Eindhoven University of Technology facilities, previously reported in [2]. Long beam shapes of one, two, three, and four layers, as illustrated in Figure 1, were extruded. Each layer had a rectangular cross section shape with 10 mm thickness and 50 mm width. The printer head was moved at 100 mm/s. The time interval between two contiguous layers was 3, 2, and 4.5 min, respectively, for elements printed with two, three, and four layers. Additionally, an extra element with two layers was printed. Especially for this element, the time interval between the two layers was 36 min. This additional two layers element was printed to investigate the time dependency of the tensile strength of the bond interface between the layers.

Both mixtures were printed in two consecutive days. After each printing section, the elements were covered with a plastic foil and pieces of wet fabric were placed in many locations underneath this foil. This strategy was adopted to make sure that the printed elements were exposed to an atmosphere with a high relative humidity therefore minimizing their eventual loss of water to the surrounding air. After 24 h of the printing session, the long printed elements were manually sawn into several pieces and stored in a curing chamber at (20 ± 2) ${}^{\circ }$C and relative humidity of (98 ± 2)% for the next 27 days.

### 2.2. Mechanical Characterization

To investigate the mechanical properties of printed composites, an extensive amount of experimental research is proposed. Through this study, the printed material’s performance was detailed and the influence of the printing process is compared with results reported in [41] for the same mixtures, traditionally cast.

#### 2.2.1. Compressive Strength Test

Compressive tests were performed in a servo hydraulic machine with a constant load rate of 2 kN/s. After 7 days of curing, cubes of 35 mm per side were mechanically sawn from the centre of the four layers’ beams and compressive strength was measured at 14 and 28 days. As an anisotropic behavior is expected due to the printing technique, the compressive load was applied perpendicular and parallel to the printing plane, corresponding to directions I and II, respectively, as defined by [43].

#### 2.2.2. Fracture Toughness Test

Specimens with dimensions of 30 × 40 × 160 mm${}^{3}$ were sawn from four layers printed beams for fracture toughness test at 28 days of curing. A notch was created in those specimens with a width of 4.5 mm and height of 7 mm for XVA3PVA20 samples and 10 mm for YVA4PVA20-S05. A three-point bending test was performed by controlling the crack mouth opening displacement (CMOD) at a rate of 0.8 μm/s until the first crack (elastic phase) and 3.3 μm/s for the rest of the test (plastic phase). Two linear variable differential transformers (LVDT) were used in the middle of the span to measure the deflection during bending.

**Figure 2:**
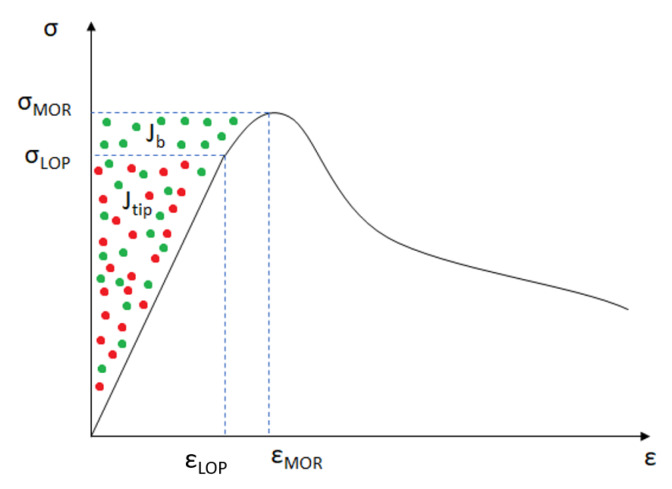
Typical crack mouth opening displacement (CMOD) curve with a representation of the calculated parameters.

The results from this test were treated to obtain several composites parameters that are useful for the mechanical performance characterization. Figure 2 exemplify these values. The following data can be extracted from the flexural stress–CMOD curves obtained from the tests:

- Limit of proportionality (LOP) and its respective CMOD value: The LOP is the flexural stress obtained from the the first point out of the linear elastic phase;
- Modulus of rupture (MOR) and its respective CMOD value: The MOR is the maximum flexural stress of the composite;
- Matrix crack tip fracture toughness ($J_{tip}$): This value gives information regarding the matrix of the composite, measuring the amount of energy needed from the material to go over the elastic phase;
  (1) $$J_{tip}=\sigma_{LOP}\epsilon_{LOP}-\int_{0}^{\epsilon_{LOP}}\sigma \left(\epsilon \right)d\epsilon$$
- The complementary energy ($J_{b}$): This is the complementary energy needed to achieve MOR of the composite. Therefore, this value gives information regarding the amount of energy needed from the fibres being pulled-out from the matrix up to the maximum flexural stress:
  (2) $$J_{b}=\sigma_{MOR}\epsilon_{MOR}-\int_{0}^{\epsilon_{MOR}}\sigma \left(\epsilon \right)d\epsilon$$

#### 2.2.3. Four-Point Bending Test

Samples with one (1 L), two (2 L), three (3 L), and four (4 L) layers were evaluated through four-point bending ageing 14 and 28 days of curing. These specimens were sawn from the printed elements to homogenize their width (approximately 40 mm) and the length (approximately 150 mm). This test was performed in an Instron 8872, applying a deflection speed of 1 mm/s. The span of the test was 120 mm and the load was applied by two metal rods spaced 40 mm from each other. Two LVDTs were used to measure the deflection in the middle of the span. During this test, the load was applied perpendicularly to the printing plane.

#### 2.2.4. Uni-Axial Tensile Test

Assuming that the printing technique would induce preferential orientation on the fibre direction, tensile tests were performed in specimens with one layer. Printed beams were sawn to create two batches of specimens which were tested after 35 days of curing. One batch had its tensile properties measured in the printing direction (LPA). These specimens had a width of 40 mm and a length of 150 mm. Another batch was tested with uni-axial tensile set-up with the load applied perpendicular to the printing direction (LPE). These specimens had a width of 35 mm and a length of 30 mm.

With the help of a LVDT sensors, the uni-axial tensile test was carried out in displacement control with loading rates of 1 and 0.2 μm/s respectively for samples in LPA and LPE. These two rates correspond to the same deformation ratio of 6.67 μstrain/s. To avoid stress concentrations or eccentricities due to clamping, each specimen was glued to tensile test setup with the help of a two component fast setting glue.

Specimens undergoing tensile testing had their front surface prepared for digital image correlation (DIC) analysis. A layer of white paint was applied on the front surface of the sample and randomly distributed black dots were made with a permanent marker. This pattern helps enhancing the contrast needed for the DIC software to calculate the displacements during test. The open source software Ncorr2 was employed for the DIC analysis [44] with a subset radius of 57 and subset space of 1 pixel. For each processing core used in the displacement calculations, the software methodology requires the positioning of a “seed” to mark the area of the initial picture. For each sample, the best “seed” positioning shall be found based on trial and error; nevertheless, the total number of “seeds” is always the same. As soon as their positioning leads to the calculation convergence and the subset radius and space are kept the same, the results shall not be different from each other. A Canon EOS 6D camera with a 28–75 mm Tamron aspherical lens was employed to obtain a frame every two seconds. An approximate resolution of 48 μm/pixel was obtained.

#### 2.2.5. Uni-Axial Tensile Test of Two Printed Layers

As reported elsewhere [43,45,46], bond interface between printed layers can be the weakest region on the composite. The time interval between two layers, which amongst others is a function of the object size and print nozzle speed, is crucial [43,46]. When a critical threshold time interval is exceeded, a so-called cold-joint may be formed leading to a significantly reduced strength compared to the bulk material. This phenomenon is well known in the field of concrete repair [47,48]. Recommendations to avoid problems in this area usually point out larger surface contact employing grooved surfaces and substrate pre-wetting to enhance the bond between both materials. Most of the techniques cited before have not yet been approached within the 3D printing context. The application of an articulated interlock has been successfully applied by Zareyian et al. [49] to significantly increase the bond strength.

In order to study the mechanical performance of the interface of two printed layers, elements with two layers were printed with the second layer being placed within two different time intervals (3 and 36 min). Afterwards, the specimens were sawn from such elements in order to create prismatic specimens sized 10 × 10 × 20 mm${}^{3}$. At the age of 28 days, these specimens were tested applying a tensile load perpendicular to the interface plane between two printed layers. The deformation was at 0.1 μm/s by means of LVDT sensors. This rate corresponds to a strain rate of 5 μstrain/s. As well as described for the LPA and LPE tests, each sample was also glued in the tensile test setup. Through this test, the interface bond strength could be assessed, and a failure zone could be identified.

### 2.3. Air Void Content and Fibre Orientation Assessment

To allow a more extensive interpretation of the results of the mechanical tests described in the subsequent subsections, non-destructive scanning was performed to determine the air void content and fibre orientation in the printed composite. The interface area where two layers meet is the most interesting region in this kind of composite. Several studies have used non-destructive methods to study the local microstructure [46], to relate growing layer interval times to increased porosity [50], and larger capillary water ingress [51].

To evaluate the anisotropic behavior of printed fibre reinforced cementitious materials, X-ray computerized micro tomography (X-ray CT) was performed by means of a Phoenix Nanotom X-ray. X-ray CT was performed on prismatic specimens with dimensions 10 × 10 × 20 mm${}^{3}$, sawn from two-layers printed beams of each studied mixture. A notch was made in the sample to indicate the printing direction. The reconstructed scanned volumes achieved a resolution of 7.5 μm/pixel. 3D reconstruction of the acquired radiographs was done through the software VG Studio (Version 3.3, Volume Graphics).

The reconstructed 3D volume was then analysed with the help of Trainable Weka Segmentation tool [52] from FIJI [53]. Two different segmentation procedures were implemented in order to (1) quantify the air voids and (2) to qualitatively and quantitatively assess fibre orientation in the composite.

The latter segmentation was carried out only on two randomly selected representative elementary volume (REV) extracted from each of the layers of the reconstructed volume. The REVs were composed by two stacks of 75 pictures, one in each printed layer. Therefore, in total, four volumes consisting of a minimum of approximately 150 mm${}^{3}$ were analysed per mixture. The latter segmentation distinguished between four phases, namely: air voids, bulk paste, large limestone grains and finally the imprints left from the fibres in the matrix. The segmented REVs were transformed into binary images to further distinguish between fibre imprints and the other phases. The segmented fibres were further filtered by constraining the individual fibre imprints to have at least an area of 10 pixel${}^{2}$ and a low circularity, between 0 and 0.3. To determine the orientation of a given fibre, the Feret’s angle was used. This angle is given in a range from 0 to 180${}^{\circ }$. According with the sample preparation, horizontal fibres (angles of 0 or 180${}^{\circ }$) represent total alignment with the printing direction, and vertical fibres (90${}^{\circ }$) represent total perpendicular alignment to the printing direction. These results were used to investigate the influence of the extrusion of the material on the final fibre orientation of the printed composite.

## 3. Results

### 3.1. Compressive Strength

The results obtained from the compression test were summarized in Table 2. Although slight differences in compressive strength between loading directions in relation to the printing plane were observed for both compositions, they are rather small (between 5 and 15 %). For the X series, the difference is less than the standard deviation, for the Y series somewhat larger. This seems to be more or less in line with results reported in literature: either a limited [54] or no directional dependency was found [43,55]. This indicates as well that the eventual flaws generated by the printing technique does not influence the performance of this mechanical property. The compressive strength of these printed specimens was also found to be close to the compressive strength of cast specimens, as presented in [41]. As Wolfs et al. [43] reported a significant strength reduction for printed specimens compared to the cast ones, it seems that the dough like consistency of the proposed SHCC discussed in this study was less vulnerable to casting parameters, such as compaction.

### 3.2. Fracture Toughness Test

**Figure 3:**
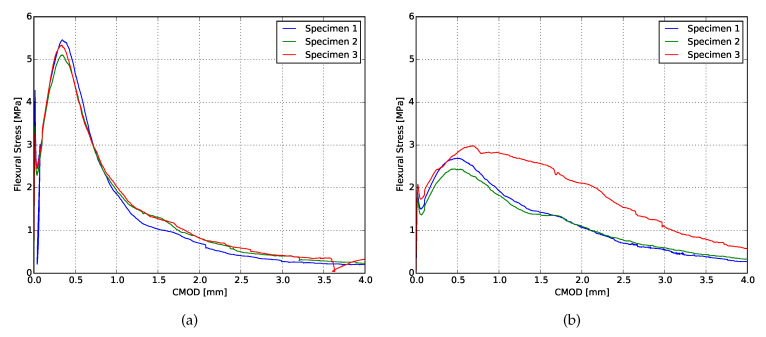
Flexural stress × CMOD curves from fracture toughness tests. (**a**) XVA3PVA20; (**b**) YVA4PVA20-S05.

The flexural stress-CMOD curves obtained from the fracture toughness test are shown in Figure 3 and the results are summarized in Table 3. Both mixtures presented ductile behavior, although mixture XVA3PVA20 achieved superior performance. According to [56,57], in order to obtain pseudo strain-hardening behavior, the composite must satisfy the condition $J_{b}$ > $J_{tip}$. As can be observed in the summarized results, the performances of both composites are in agreement with this condition. However, the XVA3PVA20 specimens exhibited considerably more stiff post-crack behavior than the YVA4PVA20-S05 specimens. This means that the fibres immediately took over the tensile stresses, which might indicate a much stronger mechanical bond between the fibre and the matrix. The large difference in the performance of both mixtures is expected since there are significant differences in the both matrices. Two differences are highlighted: the maximum aggregate particle size and the water-to-binder ratio. While XVA3PVA20 only uses limestone powder as aggregate, YVA4PVA20-S05 uses sand with maximum grain size up to 500 μm. The influence of the aggregate grain size in the ductility of SHCCs was explored before in [58]; however, the study reported not a significant change in the ductility of the SHCC when larger aggregate is used in replacement of micronized quartz. Additionally, it is also important to notice that the water-to-binder ratio of both mixtures is different (0.4 for XVA3PVA20 and 0.5 for YVA4PVA20-S05 considering only 30% of the fly ash as binder), therefore the overall mechanical performance of both composites shall be different.

### 3.3. Four-Point Bending

**Figure 4:**
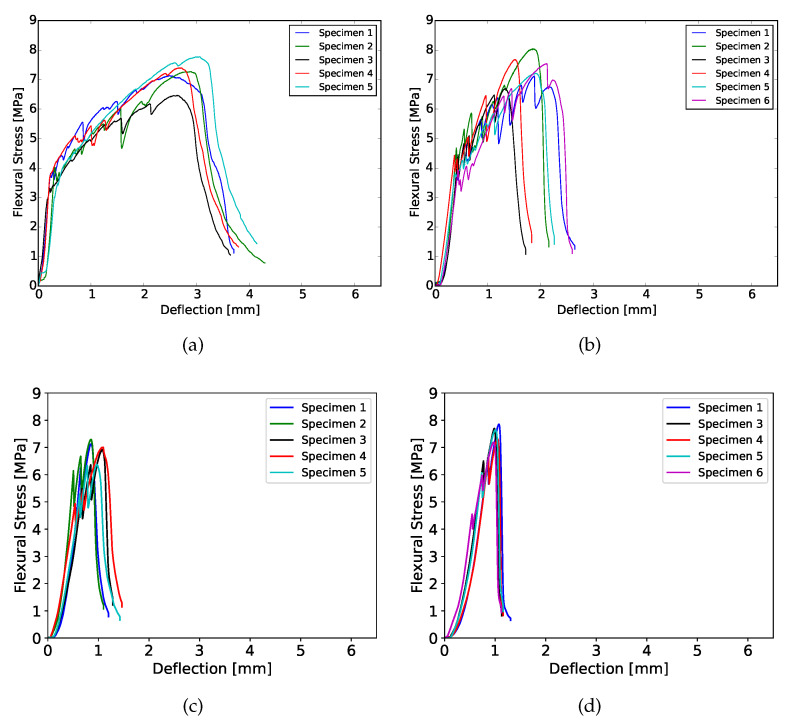
Flexural hardening behavior for XVA3PVA20 samples cured for 28 days. (**a**) 1 layer; (**b**) 2 layers; (**c**) 3 layers; (**d**) 4 layers.

**Figure 5:**
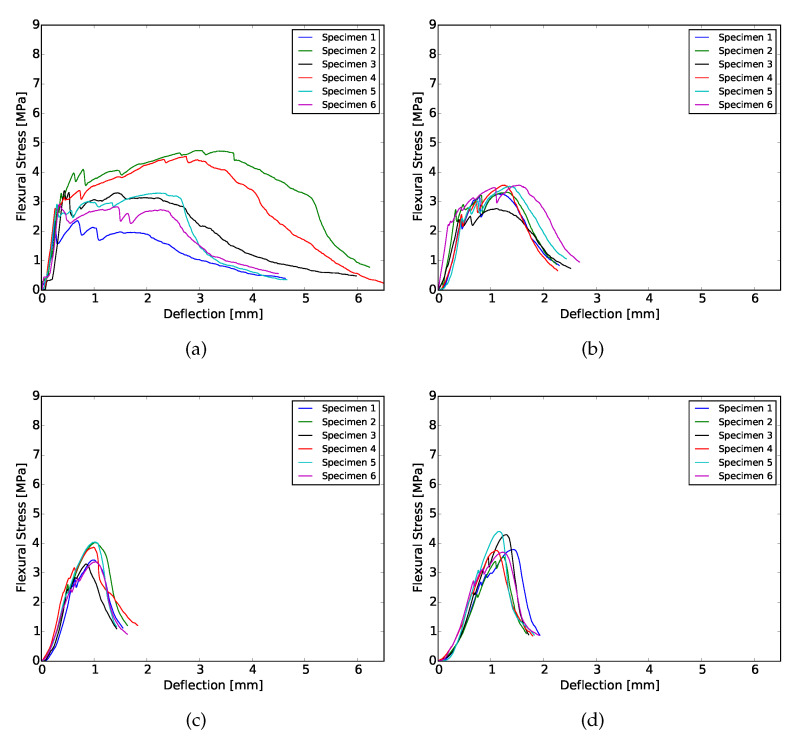
Flexural hardening behavior for YVA4PVA20-S05 samples samples cured for 28 days. (**a**) 1 layer; (**b**) 2 layers; (**c**) 3 layers; (**d**) 4 layers.

The results obtained from the four-point bending test of 28 days cured samples are shown in Figure 4 and Figure 5, as an example. At both curing ages, flexural hardening performance was obtained for all samples. Nevertheless, the results show better performance for XVA3PVA20. This indicates a lower probability to obtain strain hardening performance for composites YVA4PVA20-S05. Although, on average, YVA4PVA20-S05 specimens showed strain hardening behavior, a small number of individual specimens did not. Furthermore, no significant differences were found when the flexural performance of one-layer 3D printed samples is compared to that of conventionally cast samples [41].

**Figure 6:**
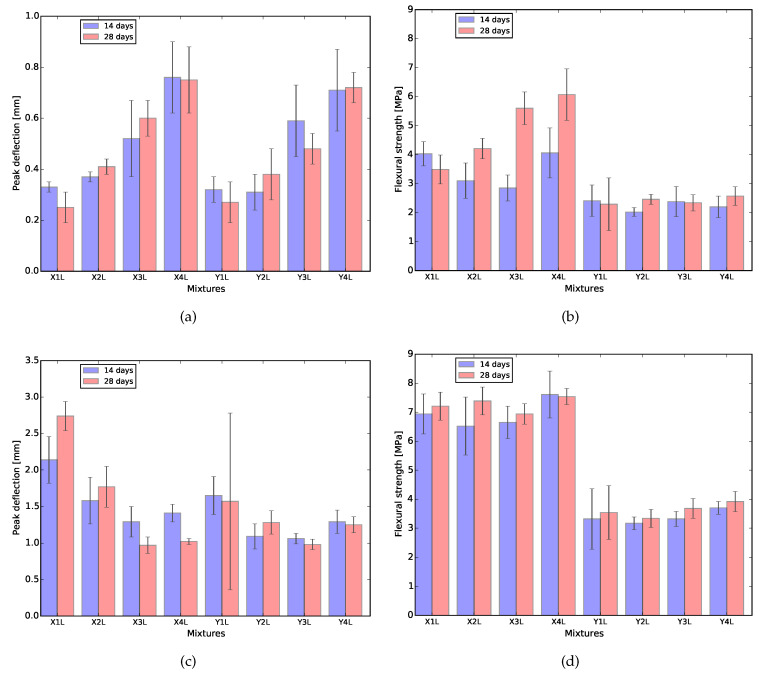
Summary of the flexural test for XVA3PVA20 (shortned to X) and YVA4PVA20-S05 (shortned to Y) samples. (**a**) deflection at first crack; (**b**) flexural strength at first crack; (**c**) deflection at maximum flexural strength; (**d**) maximum flexural strength.

As it can be observed from the flexural stress–deflection curves and also summarized in Figure 6, the number of layers of the printed element influences the flexural hardening response greatly. A lower number of cracks was registered for samples with more than one printed layer as well as lower values of deflection at the maximum flexural strength. These results indicate decreased ductility for thicker components.

This behavior is related to two main causes. The first one is purely geometrical: higher tensile stresses are generated in the tension zone due to the increased moment of inertia of the cross section. As a result, fibres are pulled-out more promptly to absorb the tensile stresses, limiting the crack propagation and the regain of flexural strength.

A second factor that plays a role is related to the printing technique employed. In order to obtain thicker elements, four layers were extruded on top of each other. This deposition generates interface regions that can be weaker, stronger or have the same mechanical properties as the bulk of the composite. In [59], the authors studied the influence in the overall mechanical performance of materials with embedded regions where properties are different from the bulk, like the interface in between layers observed in 3D printing of cementitious materials. In their study, they showed that, if the interface has better mechanical performance than the rest of the material, the total fracture crack energy increases. In the case of SHCCs, the results found by them would lead towards some loss in ductility. This is in accordance with the experimental results shown in Figure 6.

### 3.4. Uni-Axial Tensile Test

#### 3.4.1. Loading Parallel to the Printing Direction (LPA)

**Figure 7:**
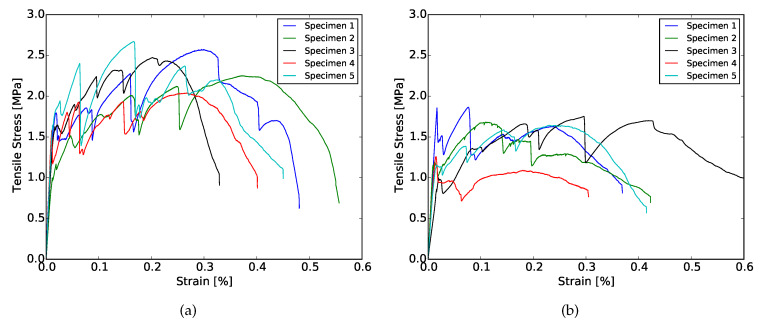
Stress–strain curves of specimens cured for 35 days resulting from uniaxial tensile tests in LPA. (**a**) XVA3PVA20; (**b**) YVA4PVA20-S05.

**Figure 8:**
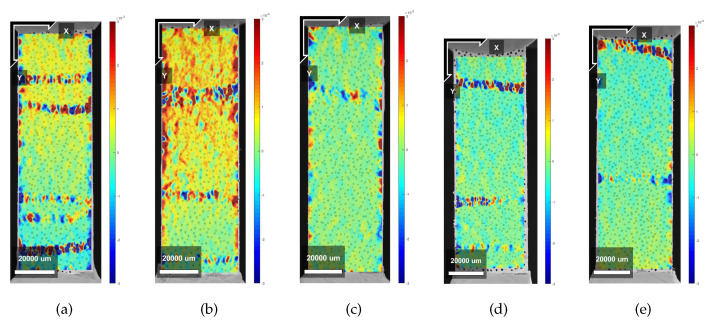
Crack pattern obtained from DIC analysis on X samples—Horizontal Deformations. (**a**) Specimen 1; (**b**) Specimen 2; (**c**) Specimen 3; (**d**) Specimen 4; (**e**) Specimen 5.

**Figure 9:**
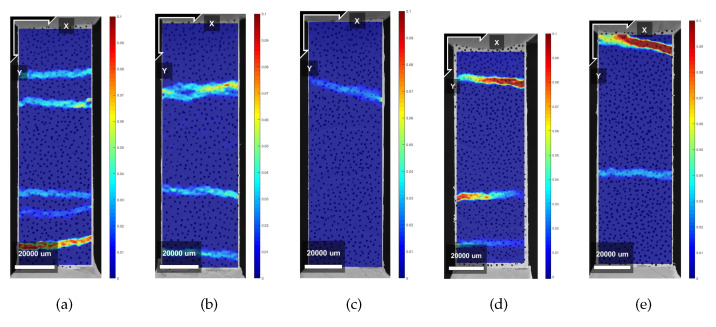
Crack pattern obtained from DIC analysis on X samples—Vertical Deformations. (**a**) Specimen 1; (**b**) Specimen 2; (**c**) Specimen 3; (**d**) Specimen 4; (**e**) Specimen 5.

**Figure 10:**
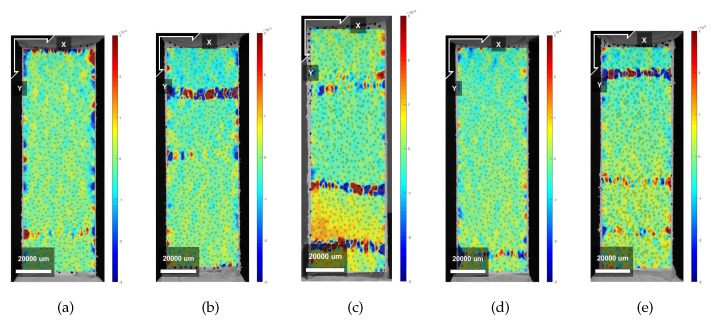
Crack pattern obtained from DIC analysis on Y samples—Horizontal Deformations. (**a**) Specimen 1; (**b**) Specimen 2; (**c**) Specimen 3; (**d**) Specimen 4; (**e**) Specimen 5.

**Figure 11:**
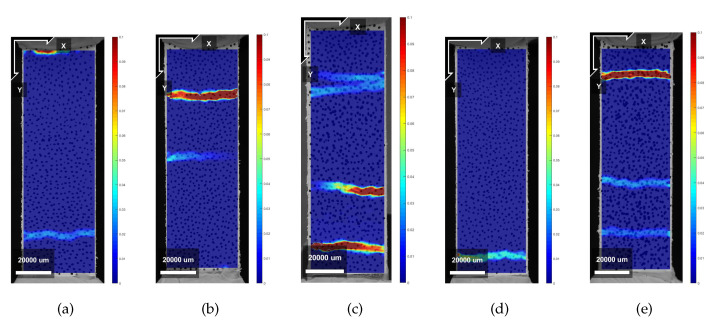
Crack pattern obtained from DIC analysis on Y samples—Vertical Deformations. (**a**) Specimen 1; (**b**) Specimen 2; (**c**) Specimen 3; (**d**) Specimen 4; (**e**) Specimen 5.

Figure 7a,b show the stress–strain curves resulting from the uniaxial tensile tests in LPA for mixture XVA3PVA20 and YVA4PVA20-S05, respectively. In Table 4 and Table 5, all the results from uniaxial tensile tests are shown, including both loading directions for both mixtures, respectively. It results in being evident that tensile behavior of the composites varied dramatically when tested in LPA or in LPE. Strain hardening behavior was obtained for XVA3PVA20 specimens, whereas only a few samples from YVA4PVA20-S05 composites could be classified as such. The large deviations found for the values of deformation at maximum tensile strength for YVA4PVA20-S05 specimens are also evidence of the better mechanical performance of XVA3PVA20. DIC results also helped to clarify this conclusion. The last picture of the test is used to illustrate the crack pattern in Figure 8, Figure 9, Figure 10 and Figure 11. These figures show the distribution of the large cracks at the end of the test. Using DIC results together with the stress–strain curves, it is possible to conclude that multiple cracking was obtained in all samples, with the exception of specimen number 4 from YVA4PVA20-S05 samples. Towards the end of the test, several large cracks are formed and usually one of them concentrates the largest displacements until reaching final failure of the composite. The mechanical performance obtained in this research are similar to what was previously reported for the same mixtures in [41].

For both series XVA3PVA20 and YVA4PVA20-S05, the total strain capacity of the composites was affected by the printing technique when compared with the original results of these composites [33,34]. Several factors might have contributed to the degradation in composites ductility, such as: mix-design modification to make these materials printable, casting, and mixing procedures. Two modifications, lower total volume of water and the use of HPMC, would generate stronger matrices due to lower capillary porosity as a consequence of the lower water/binder ratio, and the internal curing promoted by the release of arrested water in the HPMC molecules [60]. Moreover, further in this study, another important difference was also discovered and will be addressed to justify the loss in the overall composite’s ductility.

#### 3.4.2. Loading Perpendicular to the Printing Direction (LPE)

The possibility of creating preferential fibre orientation in the extruded composite is one of the most important differences of such technique when compared to conventional casting. The pressure and gradient of the materials velocity inside of the hoses have the power to change fibre direction, increasing the probability to find them longitudinally oriented towards the extrusion direction [22].

This issue is a matter of large interest of research and industry. One of the techniques used to characterize the fibre orientation is the mechanical testing itself. In the case of fibre reinforced cementitious composites, the lack of fibres in one of the directions would result in lower ductility or, especially in the case of SHCCs, the lack of strain hardening.

**Figure 12:**
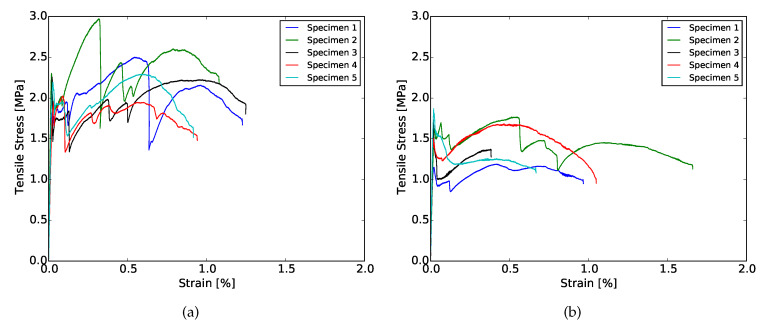
Stress–strain curves of specimens cured for 35 days resulting from uniaxial tensile tests in LPE. (**a**) XVA3PVA20; (**b**) YVA4PVA20-S05.

Figure 12a,b show the results recorded during a tensile test with LPE. Results obtained during these experiments showed that the printing technique have indeed oriented a large amount of fibres longitudinally, especially the ones found in YVA4PVA20-S05. Nevertheless, high ductility was still found for those composites. Moreover, in the case of XVA3PVA20 samples, strain hardening properties were still found. This indicates that, even if the extrusion process oriented the fibres in the mixture, the amount of reinforcement left in other directions was enough to guarantee SHCC performance. However, it is noteworthy that large deviations were found for values of deformation at maximum tensile strength for both mixtures, as seen in Table 5. This means that the preferential fibre orientation formed during the extrusion process created high anisotropy in the composite.

### 3.5. Tensile Bond Strength of Interface

**Figure 13:**
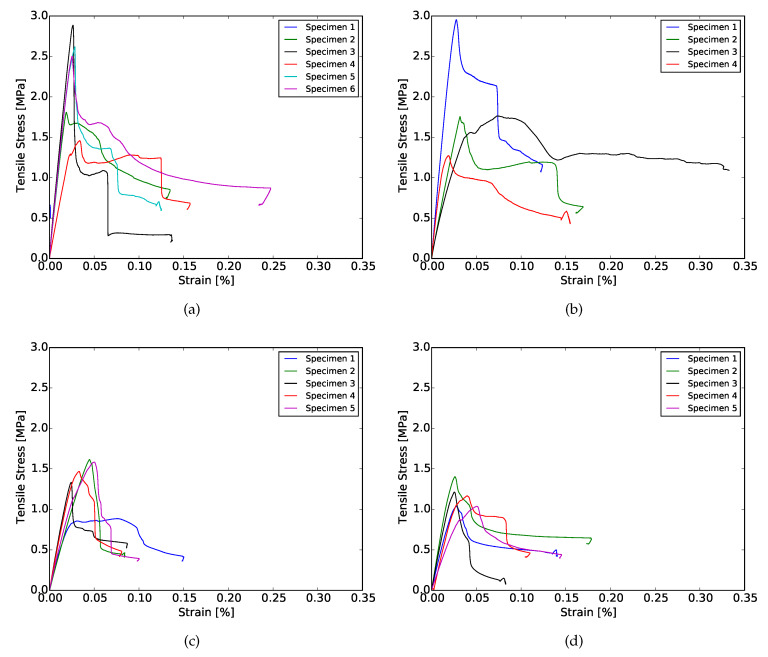
Layer interface tensile bond strength. (**a**) X2T; (**b**) X2TT; (**c**) Y2T; (**d**) Y2TT.

**Figure 14:**
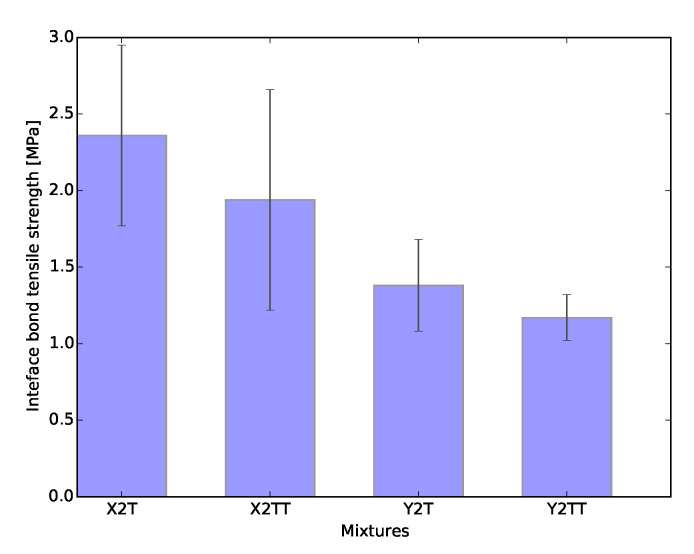
Average layer interface tensile bond strength.

Figure 13a–d show the tensile–strain curves resulted from the direct tensile tests of two printed layers of all evaluated mix-designs and time intervals. Samples named 2T had their second layer deposited after 3 min and 2TT after 36 min. The average tensile strength of these samples are also summarized in Figure 14.

The first conclusion that can be drawn from the test is that the specimens showed little ductility and no strain hardening behavior in this direction. This was expected and should be attributed to the fact that only very few fibres bridge the fracture surface. In line with the other mechanical tests, especially the uniaxial tensile tests results, the interface tensile strength of the YVA4PVA20-S05 specimens is smaller than that of the XVA3PVA20 specimens. On average, a small influence of the interlayer interval time was found, but the scatter is such that this cannot be considered significant.

The interface strength specimens with either material are comparable to or even slightly more than the tensile strength for LPA and LPE, i.e., of the bulk material. Often, it is reported that the layers’ interface tensile strength is lower than the bulk tensile strength [43,55]. In the case of the currently studied materials, this difference will be further discussed in the section where CT-scan results are discussed. As reported by [45], the nozzle height influences the bond tensile strength between printed layers. Therefore, pushing one layer against the other generates a compacted area at the interface. A visualization of the consequence of this printing technique will also be illustrated in the section where CT-scan results are discussed.

As mentioned before, the use of HPMC in the mixture might also have contributed to the enhancement of this interface region. This admixture is known to work as a water reservoir while the mixture is still in the fresh state. This property also contributes to the phenomenon known as internal curing of cementitious composites and has been investigated before [60].

These results are very important to the development of new materials and technologies for the printing of cementitious materials. Strain hardening was achieved in two directions, whereas only minor ductility was observed in the third. This confirms the intrinsic anisotropy that the technology brings.

### 3.6. CT-Scan

In order to directly investigate the particular anisotropy of the studied composites, X-ray micro CT was employed. Thus far, the results pointed out that a composite with anisotropic mechanical behavior was developed, with ductility being achieved for tensile loads applied in parallel and perpendicular to the printing direction and brittle behavior when two printed layers were pulled apart.

Comparing the results obtained from the direct tensile test of LPA, the achieved ductility was smaller than the one reported in [33]. The reason why the ductility found for those composites was below expectations can be given analysing the total air void content found in printed samples. This amount was significantly higher than what is usually found for SHCCs in literature. As these air voids can work as an obstacle to the fibre orientation, they undermine the mechanical performance of the printed composites as well. The causes of increased porosity in cementitious composites which had its rheological properties modified by HPMC were discussed along with modifications in the hydration progress of cement paste by [60].

**Figure 15:**
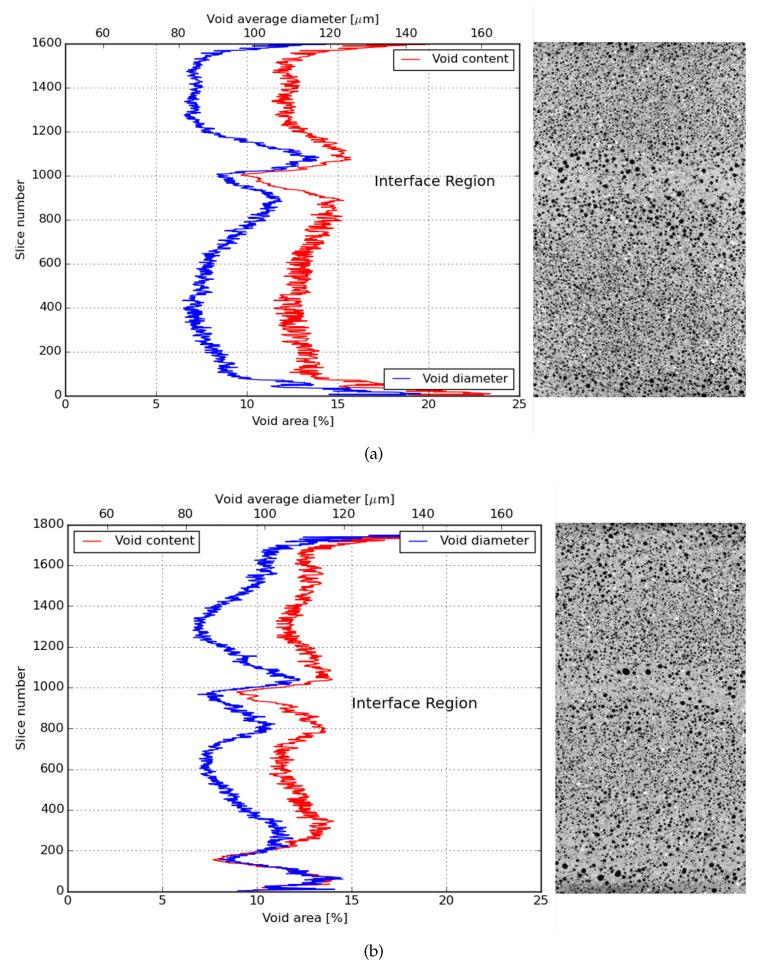
Average air void content and diameter distribution in the height of a 2-layer printed element of an X series. (**a**) X2T; (**b**) X2TT.

**Figure 16:**
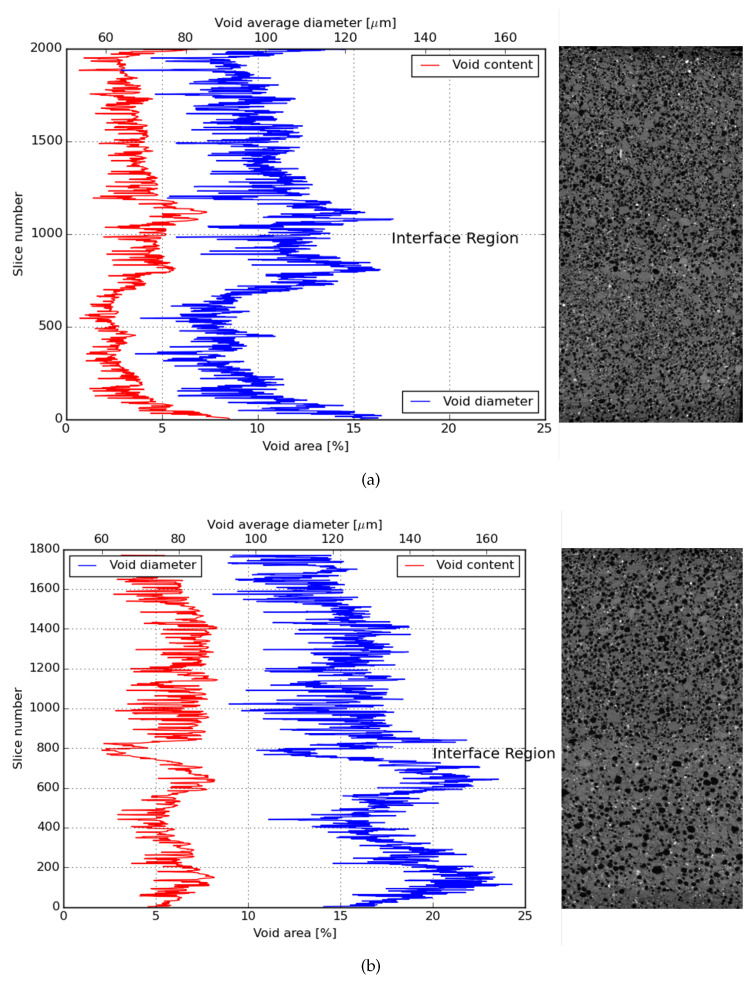
Average air void content and diameter distribution in the height of a 2-layer printed element of Y series. (**a**) Y2T; (**b**) Y2TT.

Observing the air void distribution along the height of two printed layers, another interesting result was found. From Figure 15a,b and Figure 16a,b, it was possible to visualize that the printing technique used in this investigation contributed positively to the mechanical performance of the bond strength of the interface between layers. As it can be noticed, the interface between the layers is the region with the lowest air void content in the sample. Therefore, this interface was not the weakest zone in the composite making it less probable that a crack would propagate through that zone. Nevertheless, it is also important to emphasize that the regions just under and above the interface between layers have, in general, higher air void content.

**Figure 17:**
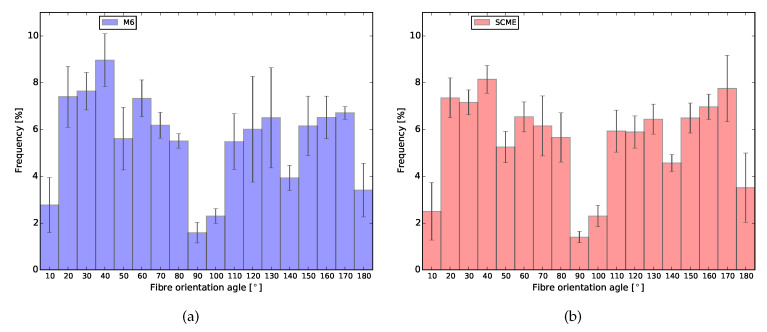
Average fibre orientation of both printed mixtures. (**a**) XVA3PVA20; (**b**) YVA4PVA20.

**Figure 18:**
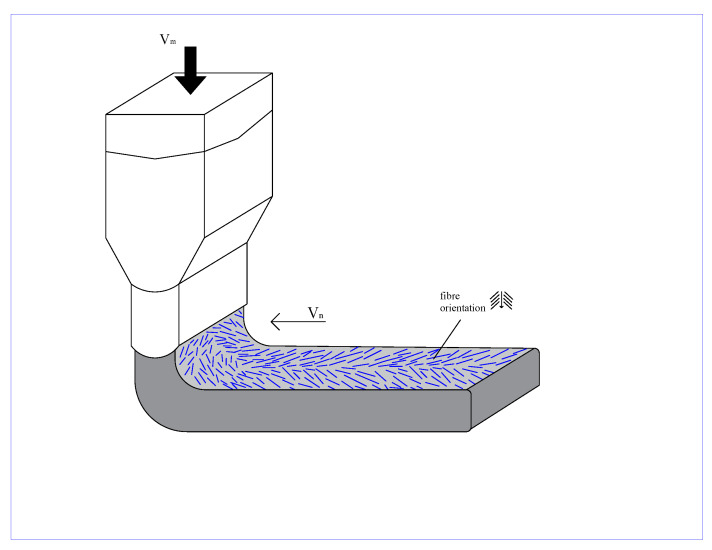
Schematic view of fibre orientation due to the printing technique.

In order to investigate the effect of air void distribution along the height of the printed beam on the fibre orientation, also the fibre orientation distribution was analysed through X-ray micro CT. Figure 17 shows the orientation of the fibres in the printed layers. The results found are in accordance with the results found in the mechanical test, especially the tensile test with LPA and LPE when ductility was found in both directions. Therefore, the extrusion process of this type of mixtures preferably oriented the fibres diagonally in relation with the printing direction. Two hypotheses are given to justify this distribution. The first one might be related to the fact that PVA fibres are flexible and the extrusion forces applied to them are too high. The second reason and most probably the strongest factor is again related to the printing technique and, to facilitate the understanding, it is also schematically represented in Figure 18. As discussed before, the printed layers are always extruded against the previous one. Therefore, the extrusion forces exert shear stresses to the printing plane causing a small spread of the material in this plane. Moreover, the combined effect of the extrusion forces and the movement of the printing nozzle generate the observed fibre preferential orientation.

## 4. Conclusions

In this experimental study, PVA-reinforced cementitious composites printed via the additive manufacturing process was extensively characterized. The methodology developed in [41] was followed for the development of printable cementitious composites. A summary of the main conclusions of the present investigations is given:

- Two types of fibre reinforced cementitious composites were successfully printed and characterized. Both demonstrated anisotropic mechanical behavior when tested in direct tensile load with ductility found in two directions loaded parallel and perpendicular to the printing direction (LPA and LPE) and brittleness in the third direction (between two printed layers);
- Both composites showed flexural and strain hardening behavior, as well as multiple cracking, in LPA. Moreover, only the samples with 0.3 wt.% of HPMC and reinforced by 2 vol.% (XVA3PVA20) showed strain hardening in LPE;
- Besides the fact that both mixtures resulted in flexural hardening, 4-point bending tests also showed that thick samples fail to deliver high ductility. This behavior is due to the larger tensile forces in the tension zone. These forces cannot be distributed homogeneously to the fibres when a crack occurs. Consequently, the energy dissipated during the cracking can not be distributed along the specimen to create multiple cracking. Further research is needed to develop ductile composites that could dissipate such high energy;
- Extruding a printed layer against the previous one is a good strategy to enhance its bond strength. As observed in this study, the interface tensile strength of both mixtures were in the same range of values of the first crack when composites were loaded in LPA and LPE. Therefore, this was not a week zone in the composite;
- The same extrusion technique that helped to improve tensile bond strength between printed layers may also have played a role in the fibre orientation, which was observed to be mainly diagonal to the print direction, rather than parallel to it. Flow speed differences in the filament also likely contribute to this fibre orientation.

## Figures and Tables

**Table 1:** Mix design summary in [kg/m${}^{3}$].

	XVA3 PVA20	YVA4 PVA20-S05
**CEM I 42.5**	259.2	480.2
**Blast Furnace Slag**	604.9	0
**Fly ash**	0	567.6
**Limestone powder**	864.1	109.1
**Sand (125–250) μm**	0	186.3
**Sand (250–500) μm**	0	294
**PVA**	26	26
**HPMC**	5.1	6.5
**Superplasticizer**	17.3	13
**Water**	345.6	327.4

**Table 2:** Compressive strength of printed samples [MPa].

	Age [days]	XVA3 PVA20	YVA4 PVA20-S05
Printed layers perpendicular to the load	14	37.63 ± 3.8	14.57 ± 0.39
	28	44.09 ± 4.33	17.66 ± 0.24
Printed layers parallel to the load	14	34.98 ± 1	12.03 ± 1.04
	28	41.93 ± 1.88	15.02 ± 0.52

**Table 3:** Summary of results obtained for the fracture toughness test.

	XVA3PVA20	YVA4PVA20-S05
**LOP [MPa]**	1.10 ± 0.17	0.56 ± 0.04
**CMOD at LOP [μm]**	10.60 ± 1.29	9.49 ± 0.93
**MOR [MPa]**	1.84 ± 0.09	0.97 ± 0.10
**CMOD at MOR [μm]**	341.99 ± 4.00	545.06 ± 112.15
***J_tip_* [kJ/m^2^]**	16.37 ± 4.51	7.11 ± 1.51
***J_b_* [kJ/m^2^]**	430.74 ± 14.62	260.94 ± 67.97

**Table 4:** Mechanical performance at the first crack of printed composites.

	Tensile Strength First Crack [MPa]	Deformation at First Crack [%]
XLPA	1.53 ± 0.23	0.023 ± 0.006
XLPE	1.84 ± 0.47	0.017 ± 0.003
YLPA	1.28 ± 0.33	0.022 ± 0.017
YLPE	1.15 ± 0.2	0.012 ± 0.003

**Table 5:** Maximum mechanical performance of printed composites.

	Maximum Tensile Strength [MPa]	Deformation at Maximum Tensile Strength [%]
XLPA	2.4 ± 0.26	0.26 ± 0.08
XLPE	2.41 ± 0.36	0.31 ± 0.26
YLPA	1.64 ± 0.23	0.15 ± 0.12
YLPE	1.65 ± 0.27	0.29 ± 0.26
